# Polysome profiling reveals broad translatome remodeling during endoplasmic reticulum (ER) stress in the pathogenic fungus *Aspergillus fumigatus*

**DOI:** 10.1186/1471-2164-15-159

**Published:** 2014-02-25

**Authors:** Karthik Krishnan, Zhaowei Ren, Liliana Losada, William C Nierman, Long Jason Lu, David S Askew

**Affiliations:** 1 Department of Pathology & Laboratory Medicine, University of Cincinnati College of Medicine, 231 Albert Sabin Way, Cincinnati, OH 45267-0529, USA; 2 Division of Biomedical Informatics, Cincinnati Children’s Hospital Research Foundation, Cincinnati, OH 45229, USA; 3 The J. Craig Venter Institute, 9704 Medical Center Drive, Rockville, MD 20850, USA; 4 Department of Pathology, University of Cincinnati, PO Box 670529, Cincinnati, OH 45267-0529, USA

**Keywords:** *Aspergillus fumigatus*, UPR, Unfolded protein response, ER stress, Translational regulation, Polysome profiling, Yvc1

## Abstract

**Background:**

The unfolded protein response (UPR) is a network of intracellular signaling pathways that supports the ability of the secretory pathway to maintain a balance between the load of proteins entering the endoplasmic reticulum (ER) and the protein folding capacity of the ER lumen. Current evidence indicates that several pathogenic fungi rely heavily on this pathway for virulence, but there is limited understanding of the mechanisms involved. The best known functional output of the UPR is transcriptional upregulation of mRNAs involved in ER homeostasis. However, this does not take into account mechanisms of translational regulation that involve differential loading of ribosomes onto mRNAs. In this study, a global analysis of transcript-specific translational regulation was performed in the pathogenic mold *Aspergillus fumigatus* to determine the nature and scope of the translational response to ER stress.

**Results:**

ER stress was induced by treating the fungus with dithiothreitol, tunicamycin, or a thermal up-shift. The mRNAs were then fractionated on the basis of ribosome occupancy into an under-translated pool (U) and a well-translated pool (W). The mRNAs were used to interrogate microarrays and the ratio of the hybridization signal (W/U) was used as an indicator of the relative translational efficiency of a mRNA under each condition. The largest category of translationally upregulated mRNAs during ER stress encoded proteins involved in translation. Components of the ergosterol and GPI anchor biosynthetic pathways also showed increased polysome association, suggesting an important role for translational regulation in membrane and cell wall homeostasis. ER stress induced limited remodeling of the secretory pathway translatome. However, a select group of transcription factors was translationally upregulated, providing a link to subsequent modification of the transcriptome. Finally, we provide evidence that one component of the ER stress translatome is a novel mRNA isoform from the *yvc1* gene that is induced by ER stress in a UPR-dependent manner.

**Conclusions:**

Together, these findings define a core set of mRNAs subject to translational control during the adaptive response to acute ER stress in *A. fumigatus* and reveal a remarkable breadth of functions that are needed to resolve ER stress in this organism.

## Background

The opportunistic mold pathogen *Aspergillus fumigatus* causes life-threatening pulmonary infections that have the potential to progress into invasive aspergillosis, a disseminated disease with a very high rate of mortality [1,2]. Infections with this fungus continue to impede the successful management of patients with hematologic malignancies or solid-organ and bone marrow transplants worldwide, accounting for the highest per person hospitalization costs of all the systemic mycoses [3-5]. The ongoing expansion of the immunosuppressed population is expected to increase the incidence of the disease, which is galvanizing studies to understand more about fungal stress response pathways that could yield novel vulnerabilities for future therapeutic targeting.

Current evidence indicates that pathogenic fungi are under endoplasmic reticulum (ER) stress in the host environment and therefore depend upon adaptive stress responses pathways to support their survival during infection [6-10]. The unfolded protein response (UPR) is the major ER stress response pathway, responsible for maintaining an ER lumenal environment that is conducive to optimal protein folding [11]. *A. fumigatus* depends upon the UPR to support the expression of clinically relevant traits such as thermotolerance, cell wall/membrane homeostasis, hypoxia adaptation, iron homeostasis, nutrient assimilation from complex substrates and antifungal drug resistance [6,7]. Similar findings have also been reported in *Cryptococcus neoformans*[8], *Candida albicans*[12], *Candida glabrata*[10], and *Alternaria brassicicola*[9], suggesting that the UPR is used by diverse fungal pathogens as a regulatory hub for the expression of multiple attributes that promote virulence in the host. The UPR is triggered in response to the accumulation of unfolded proteins, a condition that arises during infection when there is an imbalance between the level of nascent proteins entering the ER and the ability of the organelle to process that load. ER protein folding may also be perturbed by adverse conditions encountered in the host such as mammalian body temperature, oxidative stress, hypoxia and nutrient limitation [13]. The UPR counters the resulting ER stress by expanding the quantity of ER-resident chaperones and folding enzymes that are needed to help membrane and secreted proteins achieve their native conformation. The current understanding of the fungal UPR is based upon the paradigm established in the model yeast *Saccharomyces cerevisiae*[14]. The pathway is controlled by Ire1 (IreA in *A. fumigatus*), an ER-transmembrane protein that detects disturbances in the ER that lead to the accumulation of unfolded proteins. Ire1 contains a lumenal sensing domain and a cytosolic effector region that contains dual enzymes: a kinase linked to an endoribonuclease (RNase). In the absence of ER stress, Ire1p exists as an inactive monomer in association with the ER-resident chaperone, Bip (also known as Kar2p). When the folding capacity of the ER is exceeded, BiP dissociates from the lumenal domain to assist with protein folding. This triggers the activation of Ire1 by a ligand-dependent two-step mechanism in which BiP dissociation is followed by direct interaction of Ire1 with unfolded proteins [15-18]. These events elicit Ire1 oligomerization in the ER membrane, resulting in a conformational change that activates the C-terminal RNase [19,20]. The substrate of this RNAse is a cytoplasmic mRNA known as *HAC1* (*hacA* in *A. fumigatus*). The excision of an unconventional intron from the *HAC1* mRNA allows in-frame translation of the bZIP transcription factor, Hac1 (HacA in *A. fumigatus*). Hac1 re-establishes ER homeostasis by remodeling the transcriptome to enhance the protein folding capacity of the ER.

Genome-wide expression profiling has demonstrated that *A. fumigatus* responds to acute ER stress by upregulating the levels of a core group of mRNAs that encode proteins with functions that support the secretory pathway [6]. However, mRNA abundance measurements do not take translational efficiency into consideration, which is a mechanism of gene regulation that can have potent effects on protein production [21-23]. Translational regulation provides the cell with a rapid-response mechanism to fine-tune protein levels in proportion to need, and is particularly important in situations where an immediate response to an environmental stress is key for survival [24,25]. Translational regulation can be studied on a global scale by interrogating microarrays with mRNAs that have been fractionated based upon ribosome occupancy [26]. This approach is based on the fact that translationally quiescent mRNAs are sequestered within messenger ribonucleoprotein (mRNP) particles or associated with single ribosomes (monosomes), whereas actively translated mRNAs are associated with multiple ribosomes (polysomes). The hybridization of a microarray with these polysome-fractionated mRNAs can thus provide insight into how the translational efficiency of individual mRNAs is modified by environmental cues. Analogous approaches have been used to study the ER stress translatome in *S. cerevisiae* and *Aspergillus niger*[27,28]. However, a global analysis of transcript-specific translational regulation has not been performed in *A. fumigatus*. In this study, polysome fractionation of mRNA was coupled with microarray detection in order to identify changes in the translational status of the *A. fumigatus* transcriptome under conditions that perturb ER homeostasis. The findings establish a core ER stress translatome and uncover evidence for extensive translational regulation during the response of *A. fumigatus* to ER stress.

## Results & discussion

### Translatome remodeling is a major component of the ER stress response in *A. fumigatus*

**Figure 1:**
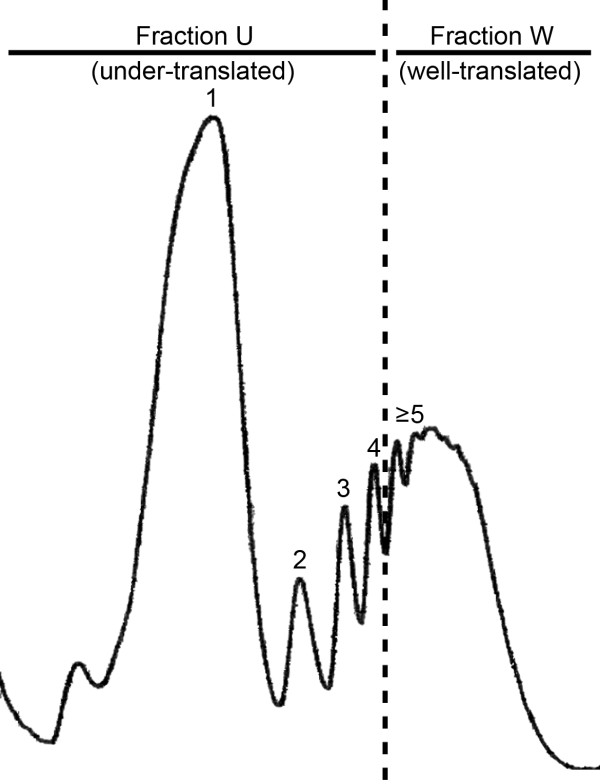
**Strategy for investigating the translational efficiency of mRNAs by polysome profiling and microarray hybridization.** A representative polysome profile shows the monosome peak (1) at the top of the gradient (left), followed by peaks representing 2,3,4 and ≥ 5 ribosomes per mRNA. Following centrifugation, the gradient was divided into two: an under-translated fraction containing mRNAs associated with 1-4 ribosomes (fraction-U) and a well translated fraction containing mRNAs associated with 5 or more ribosomes (fraction-W). The mRNAs in each fraction were then used to interrogate microarrays, as detailed in Methods. The translational efficiency of each mRNA was defined as the ratio of the hybridization signal (fraction-W/fraction-U). Those mRNAs with a higher W/U ratio during ER stress than in the absence of ER stress (using a 2-fold change between conditions as the cut-off) were considered to be subject to translational upregulation during ER stress.

The two most commonly used compounds to induce ER stress are dithiothreitol (DTT), which reduces disulfide bonds, and tunicamycin (TM), which inhibits the N-linked glycosylation that is required for optimal folding [29]. We have previously shown that treatment of *A. fumigatus* with 1 mM DTT or 10 μg/ml TM for 1 h is sufficient to trigger the UPR, defined by the induction of *hacA* splicing and a subsequent remodeling of the transcriptome to strengthen the protein folding capacity of the ER [6,7]. In the present study, we found that these conditions had undetectable effects on the overall polysome distribution in *A. fumigatus* (data not shown); both control and treated cultures showed a typical polysome profile comprised of individual ribosome subunits, the 80S monosome peak, and polysome peaks representing 2–10 ribosomes per mRNA (Figure 1). This is similar to *S. cerevisiae*, where treatment with DTT did not result in substantial reductions in global translation initiation efficiency [27]. However, it contrasts the situation in metazoans, where the analogous treatments induce global translation attenuation due to phosphorylation of eukaryotic translation initiation factor 2α (eIF2α), thereby reducing the total burden on the secretory pathway [30]. The apparent absence of a global translation attenuation response to ER stress in fungi is consistent with current evidence that the fungal kingdom lacks the ER stress sensor that controls the activation of this pathway [11,31].

**Figure 2:**
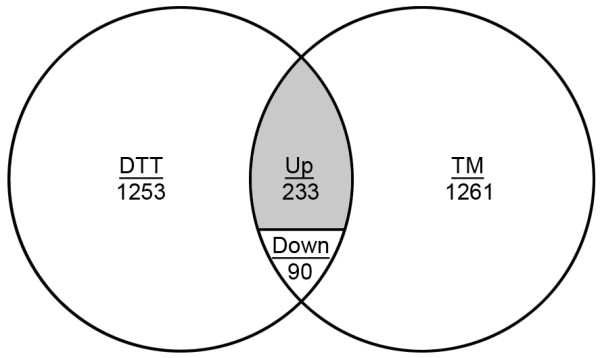
**Translationally regulated transcripts during ER stress in *A. fumigatus*.** The Venn diagram demonstrates the number of transcripts with a differential translational efficiency ratio in the presence of DTT or TM relative to untreated cultures (minimum 2-fold change in the W/U ratio). The region of overlap illustrates the number of mRNAs that are shared between the two treatments, representing mRNAs that are enriched for functions in ER stress response. The overlap contains translationally upregulated mRNAs (shaded region) as well as downregulated (Down). The area of each circle is scaled to the number of transcripts and the values for DTT and TM represent the total number of genes that were differentially regulated.

We hypothesized that a subset of mRNAs that are important to surviving ER stress would redistribute into the polysome peak to enhance their rate of translation. To test this, a genome-wide perspective of mRNA translational efficiency during ER stress was obtained by interrogating microarrays with mRNAs that were fractionated on the basis of ribosome occupancy. ER stress was induced by treating the fungus with DTT or TM, as detailed in Methods. Ribosome-associated mRNAs were then fractionated from cytoplasmic extracts into two pools: an under-translated pool (fraction-U) containing mRNAs with four or less ribosomes and a well-translated pool (fraction-W) containing mRNAs with five or more ribosomes (Figure 1). Each fraction was then used to interrogate high-density microarrays and an estimate of the translational efficiency of each mRNA was defined here as the ratio of the hybridization signal in fraction-W over that of fraction-U. Those mRNAs that showed a two-fold change (up or down) in this translational efficiency ratio during ER stress were considered to be subject to translational regulation during ER stress (see Methods for additional detail). To maximize the detection of ER stress-responsive mRNAs, and minimize chemical-specific effects, the resulting dataset was restricted to mRNAs that showed differential polysome association in response to both DTT and TM treatment. Of the 323 mRNAs that fit these criteria (Figure 2) the majority showed an increase in translational efficiency (233), suggesting that ER stress-induced translational regulation is predominantly an inductive response. It is noteworthy that the number of translationally regulated mRNAs in this analysis was over four times the size of the transcriptional response previously identified under the same conditions [6], demonstrating that remodeling of the translatome is a major component of the ER stress response in *A. fumigatus*. Validation of the microarray data was obtained by qPCR for 3 upregulated mRNAs (*erg1*, *vps55*, *and jlbA*) and one negative control (β-tubulin) (Additional file 1).

A total of 90 mRNAs showed a decrease in translational efficiency in response to DTT and TM treatment (Figure 2), 46 of which were unannotated. Analysis of the remaining 44 mRNAs using the Kyoto Encyclopedia of Genes and Genomes (KEGG) database revealed enrichment in metabolism and RNA processing categories, consistent with a shift towards more limited metabolic functions under ER stress conditions (Additional file 2). Of the 1253 mRNAs that showed altered translational efficiency in response to DTT alone, 536 were down-regulated. This contrasts a study of translational efficiency during DTT-induced stress in *A. niger*, which revealed down-regulation of 253 mRNAs and upregulation of 26 mRNAs. A direct comparison between these two datasets is difficult however, because the *A. niger* paper treated the fungus with 20 mM DTT for 2 h and fractionated mRNAs based upon occupancy with < 2 and ≥2 ribosomes [28], whereas our study used 1 mM DTT for 1 h and fractionation into pools associated with <5 and ≥5 ribosomes. The more severe ER stress conditions used in the *A. niger* study may account for the predominance of translationally repressed mRNAs in that organism, relative to the largely inductive response in *A. fumigatus*.

### ER stress induces limited remodeling of the secretory pathway translatome

The transcriptional response of *A. fumigatus* to acute ER stress is narrowly focused on upregulating the level of mRNAs that encode proteins that support the secretory pathway at multiple levels, including functions such as folding, glycosylation, ER-associated degradation, ER translocation, vesicular transport and membrane function [6]. The majority of these proteins (70%) contained predicted signal peptides that would direct them into the ER. However, only 18% of the translationally upregulated mRNAs identified in the present study contained signal peptides. Furthermore, a KEGG pathway- enrichment analysis revealed that only a minority (16%) of the mRNAs in the translationally upregulated dataset encode proteins that directly influence the secretory pathway (Table 1). In fact, none of the translationally upregulated mRNAs in this study showed increased abundance in our previous analysis of the UPR transcriptome under the identical conditions [6]. This demonstrates that the nature and scope of ER stress-induced translational regulation is fundamentally different from the transcriptional response to the same stress. Nevertheless, the identification of several transcription factor-encoding mRNAs in the translationally upregulated dataset (Table 1) suggests a mechanism whereby rapid changes in the translatome could be linked to subsequent modification of the transcriptome during ER stress. Together, these mechanisms would provide the fungus with extensive options to modify the proteome and restore ER homeostasis.

**Table 1:** List of mRNAs with increased polysome association during ER stress (treatment with DTT or TM)

**DTT**	**TM**	**mRNA**
Ribosomal proteins/translation		
2.81	2.92	60S ribosomal protein L27a (AFUA_3G05600)
2.15	3.20	40S ribosomal protein S29 (AFUA_6G12720)
2.87	2.26	40S ribosomal protein S8 (AFUA_6G07360)^#^
2.80	2.16	50S ribosomal protein L36 (AFUA_4G12810)
2.59	2.32	37S ribosomal protein S16 (AFUA_5G08350)
1.48	2.60	Mitochondrial ribosomal protein L11 (AFUA_5G11830)
2.01	1.93	Ribosome biogenesis protein (AFUA_8G04790)
1.18	2.49	37S ribosomal protein S5 (AFUA_5G11540)
1.51	1.93	40S ribosomal protein S9 (AFUA_3G06970)^#^
1.01	2.42	40S ribosomal protein Rps16 (AFUA_2G10500)^#^
1.07	2.20	60S ribosomal protein L6 (AFUA_6G09060)
4.51	2.08	Aconitate hydratase/Mitochondrial ribosomal protein subunit L49 (AFUA_3G08080)^#^
3.11	1.36	Eukaryotic translation initiation factor 3 subunit eIF-Ca (AFUA_1G05200)
1.72	2.49	Translation elongation factor eEF-1B gamma subunit, putative (AFUA_1G17120)
1.27	2.52	GTP binding protein Guf1 (AFUA_3G14350)
1.86	1.05	Eukaryotic translation initiation factor 3 subunit eIF-Ck (AFUA_3G09280)
Cell membrane/cell wall		
1.08	6.34	Squalene monooxygenase Erg1 (AFUA_5G07780)
1.61	1.43	Ergosterol biosynthesis protein Erg28 (AFUA_2G11550)
1.37	1.39	1,3-beta-glucanosyltransferase Gel2 (AFUA_6G11390)
3.51	4.88	Cell wall proline rich protein (AFUA_1G13450)
2.59	3.62	Actin cortical patch protein Sur7 (AFUA_2G02310)
4.21	2.23	CFEM domain protein (AFUA_6G14090)
1.36	1.89	GPI anchored protein (AFUA_2G07800)
1.78	1.01	GPI anchored dioxygenase (AFUA_3G01800)
1.24	3.13	Dolichol phosphate-mannose biosynthesis regulatory protein Dpm2 (AFUA_1G03020)
1.32	1.05	N-acetylglucosaminyl-phosphatidylinositol deacetylase, putative (AFUA_5G12550)
1.10	2.58	Integral membrane protein (Pth11) (AFUA_6G03600)^#^
Protein folding & modification		
1.29	2.48	Alpha-1,2-mannosyltransferase (Alg2) (AFUA_5G13210)
1.83	2.58	Disulfide isomerase (TigA) (AFUA_5G12260)^#^
1.04	2.84	Protein disulfide isomerase Pdi1 (AFUA_2G06150)
1.04	1.82	N-acetyltransferase family protein (AFUA_4G10930)
1.35	1.47	N-acetyltransferase complex ARD1 subunit (AFUA_1G09600)
2.11	2.89	Prefoldin subunit 5 (AFUA_1G10740)^#^
Endosome/protein transport and sorting		
1.71	1.94	Rho GTPase activator (Bem3) (AFUA_6G06400)
1.69	1.41	Fasciclin domain family protein (AFUA_1G14300)
3.75	1.50	Ras-like GTP-binding protein (AFUA_4G03100)
1.39	2.26	Endosomal cargo receptor (Erv14) (AFUA_6G07290)
1.58	2.16	Synaptobrevin-like protein Sybl1 (AFUA_6G11270)
1.57	3.04	RAB GTPase Vps21/Ypt51 (AFUA_3G10740)^#^
5.38	2.13	Vacuolar protein sorting 55 superfamily (AFUA_6G04780)^#^
1.03	2.77	AP-1 adaptor complex subunit sigma (AFUA_2G01570)
1.93	1.75	Mitochondrial import inner membrane translocase subunit (TIM22) (AFUA_5G02200)^#^
Transcription		
3.07	3.04	bZIP transcription factor JlbA/IDI-4 (AFUA_5G01650)^#^
1.88	3.90	CBF/NF-Y family transcription factor (AFUA_2G14250)
4.34	1.41	C6 transcription factor (AFUA_3G09130)
2.03	3.35	Transcription factor RfeF (AFUA_4G10200)
1.03	4.24	CP2 transcription factor (AFUA_1G17350)
3.09	1.71	bZIP transcription factor (LziP) (AFUA_1G16460)^#^
1.84	2.39	Transcription initiation factor TFIID, 31kd subunit, putative (AFUA_1G14600)
2.18	1.29	C6 transcription factor (AFUA_6G11230)
1.48	1.08	RNA polymerase II mediator complex component Srb8, putative (AFUA_3G06250)
1.63	3.90	CHCH domain protein (AFUA_3G06370)^#^
2.44	1.48	Nitrogen metabolite repression regulator NmrA (AFUA_5G02920)
Stress response		
1.24	1.99	General stress response phosphoprotein phosphatase Psr1/2 (AFUA_1G04790)^#^
4.37	3.43	Glutathione peroxidase Hyr1 (AFUA_3G12270)

Values represent log2[translational efficiency ratio], as described in Methods. ^#^Those mRNAs that were also subject to translational upregulation in the thermal stress dataset (37°C, 60 min).

### ER stress increases the translational state of the translation machinery

We found that components of the translational machinery were subject to increased polysome association in the presence of either DTT or TM (Table 1). This was somewhat surprising, since previous studies have shown downregulation of ribosome biogenesis genes in *A. niger* and *S. cerevisiae* exposed to DTT [27,28]. This discrepancy is likely to reflect the higher concentrations of DTT used in those studies, and/or species-specific differences in sensitivity to DTT. We speculate that a limited expansion of the translational apparatus is beneficial to *A. fumigatus* during ER stress because it provides a mechanism to rapidly increase the level of proteins that are needed to protect the ER from damage until the appropriate transcriptional modifications can be implemented. Since only a subset of the translational machinery was upregulated in *A. fumigatus*, a second possibility is that some of these proteins may have unrecognized ‘moonlighting’ functions that are relevant to ER stress responses, a possibility that is supported by an emerging literature on extra-ribosome functions for ribosomal proteins [32].

### ER stress induces remodeling of the cell wall and membrane translatome

**Figure 3:**
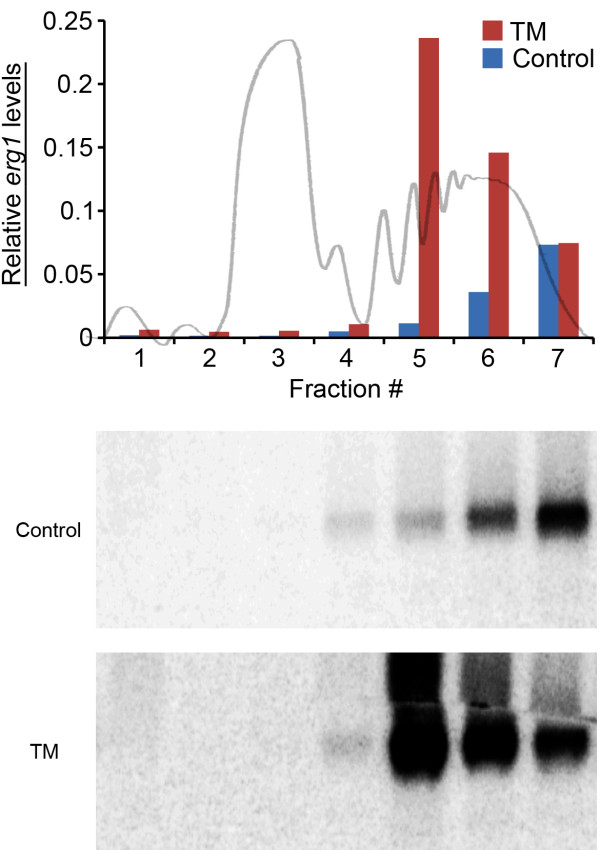
**The *erg1 *mRNA increases its association with polysomes during ER stress.** Mycelial extracts from control (untreated) and TM-treated cultures were fractionated into 7 pools. The RNA in each pool was then separated by RNA gel electrophoresis and the level of *erg1* mRNA in each fraction was determined by hybridization to an *erg1* probe. Band intensities were quantified by phosphorimager analysis and shown on the top graph. A representative OD_254_ profile is superimposed on the graph for reference. The findings demonstrate increased *erg1* mRNA levels in the polysome fraction during ER stress.

The major interface between *A. fumigatus* and the host environment is the plasma membrane and cell wall, both of which are important targets for current antifungal therapy [33]. Damage to either of these structures requires the delivery of new cell wall and membrane components to the hyphal tips, which increases the stress on the secretory pathway [7]. In this study, we found that the translational efficiency ratios for *erg1* and *erg28* mRNAs, encoding two key steps in the ergosterol pathway, increased in the presence of DTT or TM (Table 1). We have previously shown that these mRNAs do not increase in unfractionated total RNA under these same conditions [6], suggesting that they are under translational control. Northern blot analysis of polysome-fractionated RNA confirmed these findings; the amount of *erg1* mRNA that was loaded by 5 or more ribosomes was greater in the presence of DTT, indicating enhanced translation efficiency (Figure 3). It interesting to note that in addition to its enzymatic function, Erg28 has a unique role as an ER transmembrane protein that acts as a scaffold to tether other members of the ergosterol biosynthetic complex into a single functioning unit [34]. Thus, an increase in the translational efficiency of *erg28*, and potentially other ergosterol biosynthetic mRNAs, could work in concert with UPR-mediated transcriptional increases to drive flux through the sterol pathway and support membrane homeostasis. To our knowledge, this is the first evidence that mRNAs encoding ergosterol biosynthetic enzymes are subject to translational control in *A. fumigatus*. Since overexpression of mRNAs involved in sterol biosynthesis is an established mechanism of triazole antifungal drug resistance [35], it is intriguing to speculate that an increase in the translational efficiency of a mRNA in this pathway, even without a change in mRNA abundance, could represent a previously overlooked mechanism of antifungal drug resistance.

*A. fumigatus* β(1-3)glucanoxyltransferases (Gel1 and Gel2) catalyze the elongation of β(1-3) glucan side chains and influence morphogenesis and virulence [36,37]. A previous report indicates that both Gel1 and Gel2 are constitutively transcribed in *A. fumigatus*[37]. However, here we demonstrate that the translational efficiency of the *gel2* mRNA increases 2.5 fold during ER stress, suggesting that an increase in Gel2 protein is needed to protect the wall under these conditions. Gel2 contains a glycosylphosphatidylinositol (GPI) anchor that tethers it to the plasma membrane [37], which facilitates its role in maintaining cell wall integrity. Interestingly, at least three other mRNAs encoding GPI-anchored proteins of unknown function also showed increased ribosome occupancy during ER stress. In addition, ER stress caused increased polysome association of the mRNA encoding the major regulatory component for the rate-limiting step in GPI anchor biosynthesis, Dpm2, as well as the subsequent enzyme in the pathway, AfPIG-L. Together, these findings argue that rapid translation of GPI-anchored proteins is necessary to protect the fungus under conditions that disrupt ER homeostasis, mostly likely due to their role in maintaining the cell wall [37-39]. It is worth noting that GPI anchor biosynthesis is an emerging target for the development of new antifungal therapy [40-42]. Further understanding of the mechanism(s) by which translational regulation impacts GPI anchor production could suggest novel strategies to enhance pharmacologic inhibition of this pathway.

### Host-temperature adaptation involves distinct translatome remodeling

**Figure 4:**
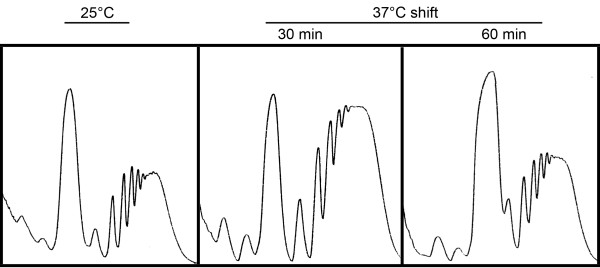
**Effects of a temperature shift on the polysome profile *A. fumigatus*.** Overnight cultures at 25°C were shifted to 37°C and polysome profiles were prepared from mycelial extracts at the indicated times.

The primary ecological niche for *A. fumigatus* in nature is composting organic material, an environment that undergoes constant fluctuations in temperature as a consequence of complex microbial activity. *A. fumigatus* has evolved mechanisms to thrive under these conditions, and is considered one of the most thermotolerant species of mold [43]. Since elevated temperatures induce conformational changes in proteins [44], an increase in temperature is likely to engage pathways that are relevant to ER stress response. We therefore compared the translational efficiency of *A. fumigatus* mRNAs at 25°C, representing the environment, to that of mRNAs following a shift to 37°C, reflecting adaptation to the mammalian host. Ribosome fractionation showed that total polysome levels increased within 30 min of the shift to 37°C, consistent with the need for increased proteins at this optimal growth temperature (Figure 4). Polysome peak heights declined somewhat after 60 min at 37°C, presumably reflecting a return to steady-state levels at the new temperature. Two criteria were employed to define differentially translated mRNAs during this transition. First, we considered all mRNAs that shifted from fraction-U to fraction-W following the temperature shift to have a temperature-induced increase in translational efficiency (two-fold cutoff). This resulted in the identification of 311 translationally upregulated mRNAs 30 min after the temperature shift, and a total of 499 mRNAs at the 1 h time-point. Some of these mRNAs could also be upregulated at the level of transcript abundance during ER stress. Thus, in order to enrich for mRNAs that are predominantly regulated at the level of translational efficiency, the dataset was narrowed to those mRNAs that showed a minimal two-fold increase in translational efficiency ratio when normalized to relative transcript abundance in unfractionated RNA. Applying these criteria, 78% of mRNAs were translationally upregulated at the 30 min time-point and 75% were upregulated at the 1 h time-point. These findings demonstrate that thermal stress is similar to DTT- and TM-induced ER stress in its reliance on translational regulation as a rapid-response mechanism to manufacture essential proteins that are needed to protect the fungus during host-temperature adaptation.

**Figure 5:**
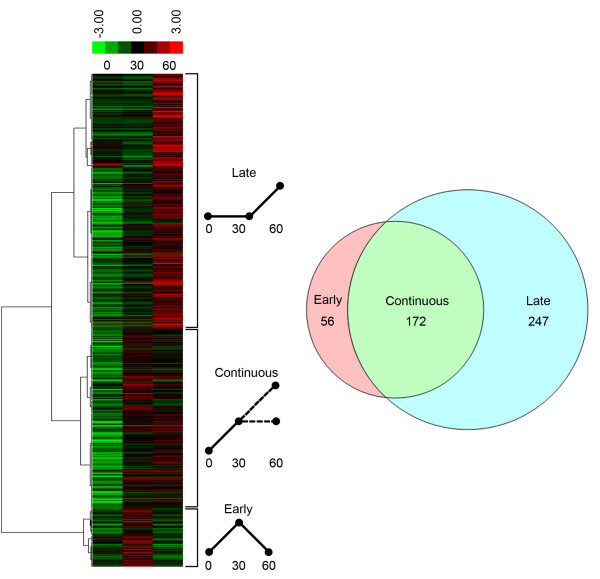
**Translational response to thermal stress.** Hierarchical clustering of mRNAs that showed temperature-dependent increases in translational efficiency fell into three major clusters (left). A schematic representation of each pattern of translational efficiency change is shown in the center: *Late* (increased at 60 min), *Continuous* (increased at 30 min and maintained or further increased at 60 min, and *Early* (transient increase at 30 min). The Venn diagram illustrates the number of mRNAs in each category.

Hierarchical clustering of all mRNAs that showed temperature-dependent increases in translational efficiency fell into three major clusters (Figure 5). The first group (‘early’) showed a transient increase in translational efficiency at 30 min that returned to baseline levels by 1 h. The second group (‘late’) showed baseline levels at 30 min but an increase at 1 h. The third group (‘continuous’) showed an increase at 30 min that was sustained at 1 h or subject to a further increase. Over-represented functional groups in the entire dataset of translationally upregulated mRNAs at 37°C included nucleotide metabolism (28), ribosome function (18), oxidative phosphorylation (26), TCA cycle (8), cell cycle (23), and secondary metabolism (18) (Additional file 3). The increased translation of mRNAs encoding proteins with roles in metabolism following the temperature shift is consistent with the fact that *A. fumigatus* grows more rapidly at 37°C than it does at 25°C. However, some metabolic genes were also enriched in the downregulated category (see the full dataset, ArrayExpress accession E-MTAB-2027), indicating that complex metabolic adjustments are operational during the transition from 25°C to 37°C. Interestingly, we found that mRNAs encoding heat-shock proteins were largely absent from the dataset of translationally upregulated mRNAs following the shift from 30°C to 37°C. However, this is congruent with previous transcriptomic analyses, which showed little upregulation of heat-shock protein-encoding mRNAs under these conditions [45,46]. We speculate that the transition from 30°C to 37°C is a relatively mild stress for this thermotolerant fungus, requiring minimal upregulation of heat-shock proteins for protection, either at the transcriptional or translational levels.

**Figure 6:**
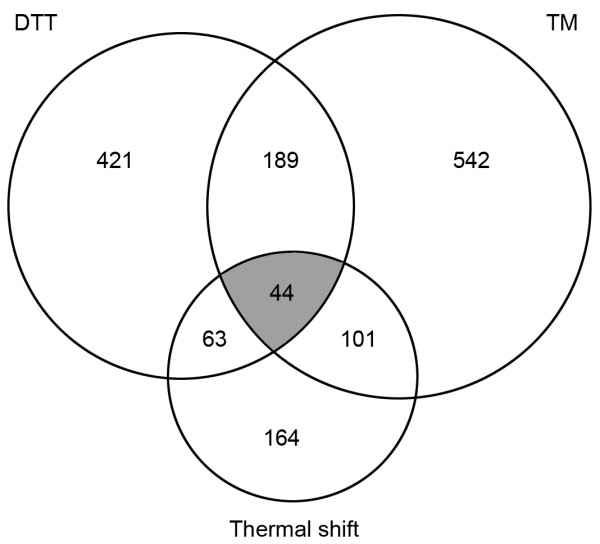
**Summary of translationally upregulated mRNAs.** The Venn diagram shows the number of transcripts with a higher translational efficiency (2-fold change) when treated with DTT or TM or when moved from 25°C to 37°C for 1 h. The overlapping region (shaded) represents a core set of translationally up-regulated genes that respond to all three types of stress in *A. fumigatus*.

The Venn diagram in Figure 6 illustrates the overlap between the transcripts that were translationally upregulated in response to the 3 types of ER stress applied in this study. A total of 44 mRNAs were shared between the three datasets, only one of which encoded a classical ER stress response protein involved in protein folding, TigA (Additional file 4). These findings demonstrate the remarkable breadth of functions that underpin ER homeostatic functions in this organism.

### The ER stress translatome contains a UPR-dependent mRNA isoform

**Figure 7:**
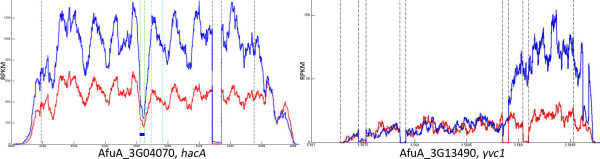
**RNA-seq coverage plots for the *hacA *and *yvc1 *mRNAs.** The number of sequence reads on the y-axis (reads per kilobase per million) is shown along the length of each gene in the absence (red) or presence (blue) of ER stress (1 mM DTT, 1 h). Vertical lines demarcate predicted intron boundaries (shown in green for the unconventional intron in *hacA*). The coverage plot for *yvc1* shows an increase in reads at the 3′ end of the gene specifically in the presence of ER stress.

The regulation of mRNA translation efficiency can occur by a variety of mechanisms, one of which includes alterations in the mRNA sequence that enhance translatability [47]. To determine whether ER stress induces changes in mRNA structure, whole transcriptome shotgun sequencing (RNA-seq) was performed in the presence or absence of DTT. The RNA-seq coverage plot for the *hacA* mRNA served as a control for this analysis, revealing splicing of the same conventional and unconventional introns that have been validated experimentally [7] (Figure 7). A substantial amount of unconventional intron splicing was evident in the absence of DTT, consistent with our previous finding that *A. fumigatus* relies upon the UPR even during normal vegetative growth [6]. This contrasts the situation in *S. cerevisiae*, where there is minimal splicing of *HAC1* in the absence of exogenous ER stress [27], possibly due to the decreased demands on the secretory pathway in a unicellular yeast. As expected, treatment of *A. fumigatus* with DTT was associated with an increase in reads across the length of the *hacA* gene (Figure 7), with the exception of the unconventional intron, demonstrating an increase in the ratio of spliced to unspliced *hacA* RNA under these conditions. The unconventional intron in *A. fumigatus hacA* is only 20 nucleotides in length, which is similar to what has been reported in the human homolog but strikingly different from the 252 nucleotide intron in the *S. cerevisiae* homolog. In *S. cerevisiae*, the unconventional intron blocks translation of the mRNA by forming a stem-loop structure with the 5’UTR [48]. The removal of the intron by Ire1-mediated splicing releases this translation block, allowing the spliced mRNA to be translated. The small size of the *hacA* intron in *A. fumigatus* makes a similar translation block mechanism unlikely, similar to what has been reported in mammals, *Caenorhabditis elegans, Candida albicans*, and other filamentous fungi [12,49-52]. In fact, the unspliced mRNA in humans is translated into a protein product that contains a hydrophobic segment that tethers the mRNA to the ER membrane, thereby facilitating splicing by Ire1 [53]. In *A. fumigatus*, both the unspliced and spliced *hacA* mRNAs can be readily identified in fraction-W by RT-PCR (data not shown), suggesting the possibility that the unspliced RNA is translated. It will be interesting to determine whether its putative encoded product is involved in a similar ER membrane tethering mechanism in *A. fumigatus*.

**Figure 8:**
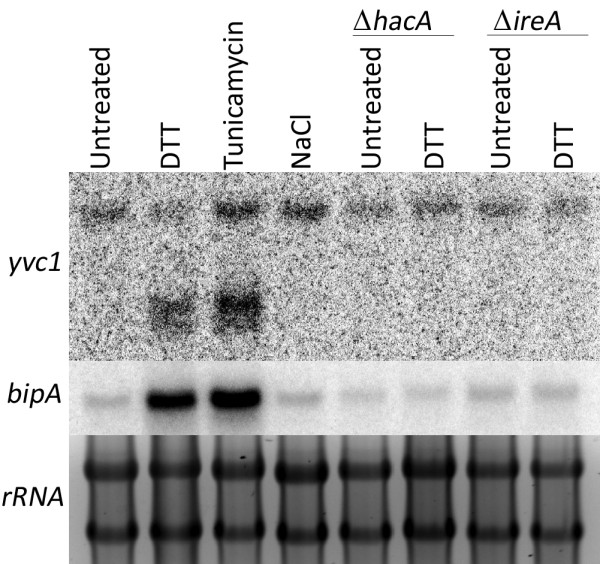
**Northern blot analysis of *yvc1 *expression.** RNA was isolated from the indicated strains in the presence or absence of ER stress (1 mM DTT for 1 h, 10 μg/ml TM for 1 h) or osmotic stress (0.8 M NaCl for 1 h). The blot was hybridized with the full-length *A. fumigatus yvc1* gene, then stripped and re-probed with the UPR marker *bipA*. Accuracy of RNA loading is shown by SYBR-green staining of rRNA.

We next analyzed the RNA-seq profiles of all 233 translationally upregulated mRNAs identified in our ER stress study (Figure 2). The RNA-seq coverage plot of the mRNA encoded by AfuA_3G13490 showed a striking change in the presence of DTT (Figure 7). This mRNA encodes the *A. fumigatus* homolog of yeast Yvc1, a transient receptor potential (TRP) channel protein in the vacuolar membrane that is the major release mechanism for intracellular calcium stores [54]. In the absence of DTT, the number of sequence reads was comparable along the length of the *yvc1* mRNA (Figure 7, red tracing), with the exception of 4 predicted introns denoted by the vertical columns. However, DTT treatment induced an increase in sequence reads, but only at the 3’-end of the gene (Figure 7, blue tracing). This mRNA did not splice out introns 3 and 4, suggesting that DTT stress was inducing a novel mRNA isoform derived from the *yvc1* transcription unit, henceforth referred to as *yvc1a*. Northern blot analysis using the full-length *yvc1* open reading frame (orf) as a probe confirmed that ER stress induced *yvc1a* expression, but osmotic stress with NaCl did not (Figure 8). In addition, DTT failed to induce *yvc1a* in two UPR mutants, Δ*ireA* and Δ*hacA*, indicating that its presence is both ER stress-specific and downstream of the UPR. Sequence analysis of the *yvc1a* cDNA identified a single long open reading frame that would encode the C-terminal 127 amino acids of the full-length Yvc1 protein (accession #: XP_001481630.1). Although the oligonucleotide used for microarray hybridization would not distinguish *yvc1a* from *yvc1*, RT-PCR analysis confirmed that both mRNAs are located in fraction-W during ER stress (data not shown), suggesting that both of them contribute to the ER stress response. No alternative *YVC1* isoforms could be identified in *S. cerevisiae* treated with either DTT or TM (data not shown), suggesting a fundamental difference between fungal genera that may reflect the increased complexity of ER stress mechanisms among filamentous fungi. Since yeast Yvc1 has a role in calcium homeostasis [54], it is appealing to speculate that the alternative isoform of this protein provides a novel regulatory mechanism to link ER stress with calcium signaling. The precise nature of the protein product encoded by this novel mRNA, its contribution to the biology of *A. fumigatus,* and the molecular mechanisms that enhance its ribosome occupancy during ER stress are currently under active investigation.

## Conclusions

In summary, this study reports the first global analysis of transcript-specific translational regulation during ER stress in the pathogenic mold *A. fumigatus*. The results define a core ER stress translatome and demonstrate that translational regulation is a major component of the response of *A. fumigatus* to environmental conditions that perturb ER homeostasis. We also provide evidence that the ER stress translatome contains a previously unidentified mRNA that is induced by ER stress in a UPR-dependent manner. Together with our previous analysis of the UPR transcriptome, these findings begin to develop a comprehensive understanding of how *A. fumigatus* responds to ER stress. Since ER stress is experienced by several fungal pathogens in the host environment [6-10], further understanding of these pathways, and the mechanisms used to deploy them, may provide a new perspective on fungal pathogenesis and offer novel strategies for therapeutic intervention.

## Methods

### Strains and culture conditions

The wt strain used in this study is AfS28 (Δ*akuA*::*ptrA*) [55]. Conidia were harvested from colonies grown on OSM plates (*Aspergillus* minimal medium containing 10 mM ammonium tartrate and osmotically stabilized with 1.2 M sorbitol). For analysis of the ER stress response, a 250 ml flask containing 50 ml of YG medium (0.5% yeast extract, 2% glucose) was inoculated with 5 X 10^7^ conidia and incubated for 16 h with shaking (200 rpm) at 37°C. ER stress was then induced by treating the cultures with 1 mM DTT or 10 μg/ml TM for 1 h. Hyperosmotic stress was induced by adding NaCl to a final concentration of 0.8 M and continuing the incubation for an additional hour. For analysis of the heat-shock response, 250 ml flasks containing 50 ml of YG medium were inoculated with 5 × 10^7^ conidia and incubated for 16 h with shaking (200 rpm) at room temperature. Thermal stress was then applied by transferring the flasks to a shaking 37°C incubator (200 rpm) and harvesting the mycelium after 30 min and 60 min of incubation.

### Polysome fractionation and RNA extraction

Sample preparation and polysome analysis was performed as previously described, with modifications [56]. Following the induction of ER or thermal stress in the cultures, translating polyribosomes were halted on the mRNAs by the addition of cycloheximide to 0.1 mg/ml and incubating at 37°C for an additional 5 min. The culture was then chilled in an ice bath for 5 min prior to harvesting the mycelium. The hyphae were washed twice with 5 ml of lysis buffer (10 mM Tris-HCl [pH 7.5], 100 mM NaCl, 30 mM MgCl_2_, 0.1 mg/ml cycloheximide, and 0.2 mg/ml heparin), flash frozen in liquid nitrogen and mechanically crushed. After resuspending in 0.5 ml of lysis buffer, the lysate was cleared by two subsequent microcentrifugation steps (15,000 x g, for 5 min at 4°C) and the RNA content in the supernatant was quantified by absorbance at 260 nm. Equal amounts of RNA (20-30 A_260_ units) were loaded onto a 12-ml linear sucrose gradient (7 - 47%) prepared in gradient buffer (50 mM Tris-acetate, 50 mM NH_4_Cl, 12 mM MgCl_2_, 1 mM DTT, and 0.2 mg/ml heparin). The gradients were centrifuged at 150,000 × g for 2.5 h at 4°C, using a Sorvall SW 41Ti rotor. Gradient analysis was performed using an ISCO gradient collector with continuous monitoring at 254 nm. Individual fractions were collected with a Foxy Jr. fraction collector and RNA was precipitated from 0.5 ml fractions by mixing with an equal volume of 6 M guanidine thiocyanate and 2 volumes of 100% ethanol and incubating overnight at -20°C. The RNA was pelleted, washed and resuspended using standard procedures. For microarray analysis, RNA from fractions containing less than 5 ribosomes/ mRNA (‘U’) or 5 or more ribosomes/mRNA (‘W’) were pooled and precipitated with 1.5 M LiCl, followed by washing to remove residual heparin. For northern blot analysis of *erg1* expression, the sucrose gradient was divided into seven sequential fractions representing the entire gradient, and the RNA was precipitated as indicated above. For experiments that required unfractionated RNA (unfractionated controls for the thermal shift microarray experiment, northern blot analysis of *erg1* mRNA, and RNA-seq analysis of DTT-treated cultures), the mycelium was crushed in liquid nitrogen and total RNA was extracted using the TRIZOL method [57].

### Microarray hybridization

The RNA labeling reactions and hybridizations were performed as described in the J. Craig Venter Institute (JCVI) standard operating procedure http://pfgrc.jcvi.org/index.php/microarray/protocols.html) and transcriptional profiles were generated by interrogating the Af293 spotted oligonucleotide microarray containing 10, 503 spots. Each gene was present in triplicate on the array, and all hybridizations were repeated in dye swap experiments. The data for each gene were averaged from the triplicate genes on each array and the duplicate dye swap experiment (a total of six readings for each gene) and the gene expression ratios were log2-transformed. Plotting open reading frame length against fold increase in the W fraction showed no bias towards longer transcripts, indicating that an increase in ribosome loading on a particular transcript is not an artifact of mRNA length (data not shown).

Functional annotation of genes present within the dataset was analyzed using FungiFun [58] and enrichment of functional groups was performed using FunCat method. Hierarchical clustering was performed using Cluster 3.0 [59] and the cluster tree was visualized using JAVA Treeview [60]. All RNA samples were hybridized with a reference sample obtained from Af293 in order to allow for cross-comparison. The translational efficiency of individual mRNAs during DTT/TM treatment was defined as the ratio of the hybridization signal in fraction-W over that of fraction-U, using a 2-fold difference between conditions as the cut-off value for a change in translational efficiency. Normalization to total mRNA abundance was not performed because the mRNAs that fit these criteria showed no increase in abundance under the same conditions [6].

The translational efficiency of individual mRNAs at 25°C and following a temperature shift to 37°C (after 30 min or 60 min) was defined as the ratio of the hybridization signal in fraction-W over that of fraction-U, using a 2-fold change between conditions as the cut-off value for a change in translational efficiency. In order to enrich for mRNAs that are predominantly regulated by changes in translational efficiency (as opposed to transcript abundance), the dataset was normalized to transcript levels in unfractionated RNA. RNA abundance was determined by interrogating the microarrays with unfractionated RNA and the change in the translational efficiency of each mRNA upon thermal shift was calculated as (fraction-W/fraction-U)/total transcript abundance.

### RNA sequencing

RNA-seq was performed by the Genomics Sequencing Core (GSC) at the University of Cincinnati. Using TruSeq RNA sample preparation kit (Illumina), total RNA (RIN ≥ 7.0, Agilent 2100 Bioanalyzer) was converted into a library of template molecules suitable for subsequent cluster generation and sequencing by Illumina HiSeq. Poly(A)_n_ mRNA was extracted and fragmented into smaller pieces (~140 nt). The cleaved RNA fragments were converted into first strand cDNA using reverse transcriptase and random primers, followed by second strand synthesis using DNA polymerase I and RNAse H. The cDNA fragments were then subject to end-repair followed by addition of a single ‘A’ base and ligation of adapters. The products were indexed individually, purified and enriched by PCR to create the final cDNA library. The generated library was validated and quantified using Kapa Library Quantification kit (Kapabiosystem). Six individually indexed cDNA libraries of equal amounts were pooled for clustering in cBot system (Illumina). Libraries were clustered onto a flow cell using Illumina’s TruSeq SR Cluster Kit v3, and sequenced for 50 cycles using TruSeq SBS kit on Illumina HiSeq system.

FASTQ files containing 50 bp single-end RNA-Seq reads were mapped to the *Aspergillus fumigatus* genome sequence (taxid:330879) by TopHat [61]. Transcript assembly and abundance estimation were performed by Cufflinks [62].

Reads corresponding to 233 genes of interest were filtered and the coverage of each nucleotide position was counted using a semi-automated method in order to ensure accuracy of analysis. Coverage plots for each of the 233 genes under two conditions were plotted using Matlab®.

### Analysis of mRNA expression by northern blot analysis and qPCR

RNA samples were fractionated by formaldehyde gel electrophoresis, and visualized by SYBR green staining. The RNA was then transferred to BioBond nylon membranes (Sigma) and hybridized to a ^32^P-labeled DNA probe as previously described [7]. Probes specific for the *A. fumigatus erg1, yvc1* and *bipA* genes were PCR amplified from genomic DNA using the following primers: *erg1*: 5′- CGTCAGTGTTGTTGAGAC-3′ and 5′- GAAGGTCGAGAGCTGCTTC-3′; *yvc1*: 5′- CAATGCTGTGGACGAGTACATG-3′ and 5′ - GTGCTCCTCTGTATCCTTCTTC-3′; *bipA*: 5′- GTCTGATTGGACGCAAGTTC-3′ and 5′- ATCTGGGAAGACAGAGTACG-3′. Hybridization intensities were quantified by phosphorimager analysis using Image Lab software.

For qPCR analysis one μg of RNA from pooled fractions corresponding to fraction-U or fraction-W was reverse-transcribed with M-MuLV reverse transcriptase (NEB) using oligo (dT)_18_ and 18S rRNA primers (primer 713-TGAGCCGATAGTCCCCCTAA and primer 714-GACTCAACACGGGGAAACTC). The qPCR was performed using the iTaq™ universal SYBR® green supermix (Bio-Rad) according to the manufacturer’s protocol. The melting curve was monitored to verify specificity of the amplification reaction. Controls reactions in the absence of reverse transcriptase were used verify the absence of DNA contamination. The 18S rRNA present within fraction-U or fraction-W was used as an endogenous control to derive a ∆Ct value for each fraction. A translational efficiency ratio (W/U) was derived by subtracting ∆Ct of fraction-W from that of fraction-U, representing ∆∆Ct. Change in W/U ratios upon treatment with DTT or TM was then plotted using 2-∆∆Ct of untreated samples as the reference. Primers used for qRT-PCR are as follows: β-tubulin (AfuA_1g10910), primer 554-CACGGATCTTGGAGATC and primer 562-ACAACTTCGTCTTCGGCCAG; squalene monooxygenase *erg1* (AfuA_5g07780), primer 810-AGCTGCGATCTATGCCGAATTCCT and primer 799-TCCCAGTTGGAAGTAACGGAAGCA; vacuolar protein sorting 55 superfamily *vps55* (AFUA_6G04780), primer 804-GCGCTCTCCTTTGTTCTTGCCATT and primer 805-AAGACCTCCGAGGATGGACATGAT; bZIP transcription factor *jlbA/IDI-4* (AFUA_5G01650), primer 813-TTGATGTGAACGACTCTCTGCCGT and primer 814-TAGCTTCGACACCCGCATCTTCAA. The data were compared by Student’s t-test and a p < 0.05 was considered significant (indicated by the asterisk, Additional file 1).

### Data availability

The microarray and RNA-seq data sets reported in this article are available in the ArrayExpress database (microarray accession E-MTAB-2027, RNA-seq accession ERP004296).

## Abbreviations

ER: Endoplasmic reticulum; UPR: Unfolded protein response; RNAse: Endoribonuclease; DTT: Dithiothreitol; TM: Tunicamycin; KEGG: Kyoto Encyclopedia of Genes and Genomes; GPI: Glycosylphosphatidylinositol; TRP: Transient receptor potential; YG: Yeast extract/glucose medium.

## Competing interests

The authors declare that they have no competing interests.

## Authors’ contributions

KK performed the polysome fractionation, RNA isolation, and drafted the manuscript. ZR and LJL performed the RNA-seq analysis. KK, LL, WCN and DSA performed the microarray hybridization analysis. DSA conceived of the study, participated in its design and coordination and helped to draft the manuscript. All authors read and approved the final manuscript.

## Supplementary Material

Additional file 1 **Validation of the translationally regulated dataset by qPCR.** The levels of 18S rRNA in fraction-U or fraction-W was used as an endogenous control to derive a ∆Ct value for each fraction. A translational efficiency ratio (W/U) was then calculated by subtracting ∆Ct of fraction-W from that of fraction-U, representing ∆∆Ct. The change in W/U ratios upon treatment with DTT or TM was then plotted using 2-∆∆Ct of untreated samples (UT) as the reference. Click here for file

Additional file 2 **List of mRNAs with decreased polysome association during ER stress (treatment with DTT or TM).** Values represent log2 [translational state efficiency], as described in Methods. Click here for file

Additional file 3 List of over-represented KEGG pathways in the dataset of translationally regulated mRNA following a shift to 37°C. Click here for file

Additional file 4 **List of mRNAs with increased polysome association during each of the three forms of ER stress: treatment with DTT, TM and thermal stress.** Values represent log2[translational efficiency ratio], as described in Methods. ^#^mRNAs subject to translational upregulation in the thermal stress dataset at 60 min. Click here for file

## References

[B1] Shoham S Marr KA Invasive fungal infections in solid organ transplant recipients Future Microbiol 2012 7 5 639 655 10.2217/fmb.12.28 22568718 PMC4222063

[B2] Segal BH Aspergillosis N Engl J Med 2009 360 18 1870 1884 10.1056/NEJMra0808853 19403905

[B3] Kim A Nicolau DP Kuti JL Hospital costs and outcomes among intravenous antifungal therapies for patients with invasive Aspergillosis in the United States Mycoses 2011 54 5 e301 312 10.1111/j.1439-0507.2010.01903.x 20557463

[B4] Wilson LS Reyes CM Stolpman M Speckman J Allen K Beney J The direct cost and incidence of systemic fungal infections Value Health 2002 5 1 26 34 10.1046/j.1524-4733.2002.51108.x 11873380

[B5] Lin SJ Schranz J Teutsch SM Aspergillosis case-fatality rate: systematic review of the literature Clin Infect Dis 2001 32 3 358 366 10.1086/318483 11170942

[B6] Feng X Krishnan K Richie DL Aimanianda V Hartl L Grahl N Powers-Fletcher MV Zhang M Fuller KK Nierman WC HacA-independent functions of the ER stress sensor IreA synergize with the canonical UPR to influence virulence traits in *Aspergillus fumigatus* PLoS Pathog 2011 7 10 e1002330 10.1371/journal.ppat.1002330 22028661 PMC3197630

[B7] Richie DL Hartl L Aimanianda V Winters MS Fuller KK Miley MD White S McCarthy JW Latge JP Feldmesser M A role for the unfolded protein response (UPR) in virulence and antifungal susceptibility in A*spergillus fumigatus* PLoS Pathog 2009 5 1 e1000258 10.1371/journal.ppat.1000258 19132084 PMC2606855

[B8] Cheon SA Jung KW Chen YL Heitman J Bahn YS Kang HA Unique evolution of the UPR pathway with a novel bZIP transcription factor, Hxl1, for controlling pathogenicity of *Cryptococcus neoformans* PLoS Pathog 2011 7 8 e1002177 10.1371/journal.ppat.1002177 21852949 PMC3154848

[B9] Joubert A Simoneau P Campion C Bataille-Simoneau N Iacomi-Vasilescu B Poupard P Francois JM Georgeault S Sellier E Guillemette T Impact of the unfolded protein response on the pathogenicity of the Neurotrophic fungus *Alternaria brassicicola* Mol Microbiol 2011 79 5 1305 1324 10.1111/j.1365-2958.2010.07522.x 21251090

[B10] Miyazaki T Nakayama H Nagayoshi Y Kakeya H Kohno S Dissection of Ire1 functions reveals stress response mechanisms uniquely evolved in *Candida glabrata* PLoS Pathog 2013 9 1 e1003160 10.1371/journal.ppat.1003160 23382685 PMC3561209

[B11] Gardner BM Pincus D Gotthardt K Gallagher CM Walter P Endoplasmic reticulum stress sensing in the unfolded protein response Cold Spring Harbor Perspect Biol 2013 5 3 a013169 10.1101/cshperspect.a013169 PMC3578356 23388626

[B12] Wimalasena TT Enjalbert B Guillemette T Plumridge A Budge S Yin Z Brown AJ Archer DB Impact of the unfolded protein response upon genome-wide expression patterns, and the role of Hac1 in the polarized growth, of *Candida albicans* Fungal Genet Biol 2008 45 9 1235 1247 10.1016/j.fgb.2008.06.001 18602013

[B13] Hartmann T Sasse C Schedler A Hasenberg M Gunzer M Krappmann S Shaping the fungal adaptome–stress responses of Aspergillus fumigatus Int J Med Microbiol 2011 301 5 408 416 10.1016/j.ijmm.2011.04.008 21565548

[B14] Kohno K Stress-sensing mechanisms in the unfolded protein response: similarities and differences between yeast and mammals J Biochem 2010 147 1 27 33 10.1093/jb/mvp196 19942680

[B15] Kimata Y Oikawa D Shimizu Y Ishiwata-Kimata Y Kohno K A role for BiP as an adjustor for the endoplasmic reticulum stress-sensing protein Ire1 J Cell Biol 2004 167 3 445 456 10.1083/jcb.200405153 15520230 PMC2172501

[B16] Gardner BM Walter P Unfolded proteins are Ire1-activating ligands that directly induce the unfolded protein response Science 2011 333 6051 1891 1894 10.1126/science.1209126 21852455 PMC3202989

[B17] Kimata Y Ishiwata-Kimata Y Ito T Hirata A Suzuki T Oikawa D Takeuchi M Kohno K Two regulatory steps of ER-stress sensor Ire1 involving its cluster formation and interaction with unfolded proteins J Cell Biol 2007 179 1 75 86 10.1083/jcb.200704166 17923530 PMC2064738

[B18] Credle JJ Finer-Moore JS Papa FR Stroud RM Walter P On the mechanism of sensing unfolded protein in the endoplasmic reticulum Proc Natl Acad Sci USA 2005 102 52 18773 18784 10.1073/pnas.0509487102 16365312 PMC1316886

[B19] Korennykh AV Egea PF Korostelev AA Finer-Moore J Zhang C Shokat KM Stroud RM Walter P The unfolded protein response signals through high-order assembly of Ire1 Nature 2009 457 7230 687 693 10.1038/nature07661 19079236 PMC2846394

[B20] Shamu CE Walter P Oligomerization and phosphorylation of the Ire1p kinase during intracellular signaling from the endoplasmic reticulum to the nucleus EMBO J 1996 15 12 3028 3039 8670804 PMC450244

[B21] Gygi SP Rochon Y Franza BR Aebersold R Correlation between protein and mRNA abundance in yeast Mol Cell Biol 1999 19 3 1720 1730 10022859 10.1128/mcb.19.3.1720 PMC83965

[B22] Washburn MP Koller A Oshiro G Ulaszek RR Plouffe D Deciu C Winzeler E Yates JR 3rd Protein pathway and complex clustering of correlated mRNA and protein expression analyses in *Saccharomyces cerevisiae* Proc Natl Acad Sci USA 2003 100 6 3107 3112 10.1073/pnas.0634629100 12626741 PMC152254

[B23] Greenbaum D Colangelo C Williams K Gerstein M Comparing protein abundance and mRNA expression levels on a genomic scale Genome Biol 2003 4 9 117 10.1186/gb-2003-4-9-117 12952525 PMC193646

[B24] Gebauer F Hentze MW Molecular mechanisms of translational control Nat Rev Mol Cell Biol 2004 5 10 827 835 10.1038/nrm1488 15459663 PMC7097087

[B25] Halbeisen RE Gerber AP Stress-dependent coordination of transcriptome and translatome in yeast PLoS Biol 2009 7 5 e1000105 19419242 10.1371/journal.pbio.1000105 PMC2675909

[B26] Beilharz TH Preiss T Translational profiling: the genome-wide measure of the nascent proteome Brief Funct Genom Proteom 2004 3 2 103 111 10.1093/bfgp/3.2.103 15355593

[B27] Payne T Hanfrey C Bishop AL Michael AJ Avery SV Archer DB Transcript-specific translational regulation in the unfolded protein response of *Saccharomyces cerevisiae* FEBS Lett 2008 582 4 503 509 10.1016/j.febslet.2008.01.009 18206654

[B28] Guillemette T van Peij NN Goosen T Lanthaler K Robson GD van den Hondel CA Stam H Archer DB Genomic analysis of the secretion stress response in the enzyme-producing cell factory *Aspergillus niger* BMC Genomics 2007 8 158 10.1186/1471-2164-8-158 17561995 PMC1894978

[B29] Back SH Schroder M Lee K Zhang K Kaufman RJ ER stress signaling by regulated splicing: IRE1/HAC1/XBP1 Methods 2005 35 4 395 416 10.1016/j.ymeth.2005.03.001 15804613

[B30] Harding HP Zhang Y Ron D Protein translation and folding are coupled by an endoplasmic-reticulum-resident kinase Nature 1999 397 6716 271 274 10.1038/16729 9930704

[B31] Korennykh A Walter P Structural basis of the unfolded protein response Ann Rev Cell Dev Biol 2012 28 251 277 10.1146/annurev-cellbio-101011-155826 23057742

[B32] Warner JR McIntosh KB How common are extraribosomal functions of ribosomal proteins? Mol Cell 2009 34 1 3 11 10.1016/j.molcel.2009.03.006 19362532 PMC2679180

[B33] Gastebois A Clavaud C Aimanianda V Latge JP *Aspergillus fumigatus*: cell wall polysaccharides, their biosynthesis and organization Future Microbiol 2009 4 5 583 595 10.2217/fmb.09.29 19492968

[B34] Mo C Bard M Erg28p is a key protein in the yeast sterol biosynthetic enzyme complex J Lipid Res 2005 46 9 1991 1998 10.1194/jlr.M500153-JLR200 15995173

[B35] Lewis RE Viale P Kontoyiannis DP The potential impact of antifungal drug resistance mechanisms on the host immune response to *Candida* Virulence 2012 3 4 368 376 10.4161/viru.20746 22722245 PMC3478239

[B36] Hartland RP Fontaine T Debeaupuis JP Simenel C Delepierre M Latge JP A novel beta-(1-3)-glucanosyltransferase from the cell wall of *Aspergillus fumigatus* J Biol Chem 1996 271 43 26843 26849 10.1074/jbc.271.43.26843 8900166

[B37] Mouyna I Morelle W Vai M Monod M Lechenne B Fontaine T Beauvais A Sarfati J Prevost MC Henry C Deletion of GEL2 encoding for a beta(1-3)glucanosyltransferase affects morphogenesis and virulence in *Aspergillus fumigatus* Mol Microbiol 2005 56 6 1675 1688 10.1111/j.1365-2958.2005.04654.x 15916615

[B38] Li H Zhou H Luo Y Ouyang H Hu H Jin C Glycosylphosphatidylinositol (GPI) anchor is required in *Aspergillus fumigatus* for morphogenesis and virulence Mol Microbiol 2007 64 4 1014 1027 10.1111/j.1365-2958.2007.05709.x 17501924

[B39] Gastebois A Fontaine T Latge JP Mouyna I beta(1-3)Glucanosyltransferase Gel4p is essential for *Aspergillus fumigatus* Eukaryot Cell 2010 9 8 1294 1298 10.1128/EC.00107-10 20543062 PMC2918925

[B40] Hata K Horii T Miyazaki M Watanabe NA Okubo M Sonoda J Nakamoto K Tanaka K Shirotori S Murai N Efficacy of oral E1210, a new broad-spectrum antifungal with a novel mechanism of action, in murine models of candidiasis, Aspergillosis, and fusariosis Antimicrob Agents Chemother 2011 55 10 4543 4551 10.1128/AAC.00366-11 21788462 PMC3187015

[B41] Miyazaki M Horii T Hata K Watanabe NA Nakamoto K Tanaka K Shirotori S Murai N Inoue S Matsukura M In vitro activity of E1210, a novel antifungal, against clinically important yeasts and molds Antimicrob Agents Chemother 2011 55 10 4652 4658 10.1128/AAC.00291-11 21825291 PMC3186989

[B42] McLellan CA Whitesell L King OD Lancaster AK Mazitschek R Lindquist S Inhibiting GPI anchor biosynthesis in fungi stresses the endoplasmic reticulum and enhances immunogenicity ACS Chem Biol 2012 7 9 1520 1528 10.1021/cb300235m 22724584

[B43] Bhabhra R Askew DS Thermotolerance and virulence of *Aspergillus fumigatus*: role of the fungal nucleolus Med Mycol 2005 43 Suppl 1 S87 S93 16110798 10.1080/13693780400029486

[B44] Matsumoto R Akama K Rakwal R Iwahashi H The stress response against denatured proteins in the deletion of cytosolic chaperones SSA1/2 is different from heat-shock response in *Saccharomyces cerevisiae* BMC Genomics 2005 6 141 10.1186/1471-2164-6-141 16209719 PMC1262714

[B45] Do JH Yamaguchi R Miyano S Exploring temporal transcription regulation structure of *Aspergillus fumigatus* in heat shock by state space model BMC Genomics 2009 10 306 10.1186/1471-2164-10-306 19586549 PMC2714559

[B46] Nierman WC Pain A Anderson MJ Wortman JR Kim HS Arroyo J Berriman M Abe K Archer DB Bermejo C Genomic sequence of the pathogenic and allergenic filamentous fungus *Aspergillus fumigatus* Nature 2005 438 7071 1151 1156 10.1038/nature04332 16372009

[B47] Mulder HJ Nikolaev I HacA-dependent transcriptional switch releases hacA mRNA from a translational block upon endoplasmic reticulum stress Eukaryot Cell 2009 8 4 665 675 10.1128/EC.00131-08 19181870 PMC2669205

[B48] Ruegsegger U Leber JH Walter P Block of HAC1 mRNA translation by long-range base pairing is released by cytoplasmic splicing upon induction of the unfolded protein response Cell 2001 107 1 103 114 10.1016/S0092-8674(01)00505-0 11595189

[B49] Hooks KB Griffiths-Jones S Conserved RNA structures in the non-canonical Hac1/Xbp1 intron RNA Biol 2011 8 4 552 556 10.4161/rna.8.4.15396 21593604 PMC3225973

[B50] Calfon M Zeng H Urano F Till JH Hubbard SR Harding HP Clark SG Ron D IRE1 couples endoplasmic reticulum load to secretory capacity by processing the XBP-1 mRNA Nature 2002 415 6867 92 96 10.1038/415092a 11780124

[B51] Mulder HJ Saloheimo M Penttila M Madrid SM The transcription factor HACA mediates the unfolded protein response in *Aspergillus niger*, and up-regulates its own transcription Mol Genet Genomics 2004 271 2 130 140 10.1007/s00438-003-0965-5 14730445

[B52] Saloheimo M Valkonen M Penttila M Activation mechanisms of the HAC1-mediated unfolded protein response in filamentous fungi Mol Microbiol 2003 47 4 1149 1161 10.1046/j.1365-2958.2003.03363.x 12581366

[B53] Yanagitani K Imagawa Y Iwawaki T Hosoda A Saito M Kimata Y Kohno K Cotranslational targeting of XBP1 protein to the membrane promotes cytoplasmic splicing of its own mRNA Mol Cell 2009 34 2 191 200 10.1016/j.molcel.2009.02.033 19394296

[B54] Denis V Cyert MS Internal Ca(2+) release in yeast is triggered by hypertonic shock and mediated by a TRP channel homologue J Cell Biol 2002 156 1 29 34 10.1083/jcb.200111004 11781332 PMC2173594

[B55] Krappmann S Sasse C Braus GH Gene targeting in Aspergillus fumigatus by homologous recombination is facilitated in a nonhomologous end- joining-deficient genetic background Eukaryot Cell 2006 5 1 212 215 10.1128/EC.5.1.212-215.2006 16400185 PMC1360265

[B56] Bhabhra R Richie DL Kim HS Nierman WC Fortwendel J Aris JP Rhodes JC Askew DS Impaired ribosome biogenesis disrupts the integration between morphogenesis and nuclear duplication during the germination of *Aspergillus fumigatus* Eukaryot Cell 2008 7 4 575 583 10.1128/EC.00412-07 18296619 PMC2292631

[B57] Chomczynski P Sacchi N Single-step method of RNA isolation by acid guanidinium thiocyanate-phenol-chloroform extraction Anal Biochem 1987 162 1 156 159 2440339 10.1006/abio.1987.9999

[B58] Priebe S Linde J Albrecht D Guthke R Brakhage AA FungiFun: a web-based application for functional categorization of fungal genes and proteins Fungal Genet Biol 2011 48 4 353 358 10.1016/j.fgb.2010.11.001 21073976

[B59] Eisen MB Spellman PT Brown PO Botstein D Cluster analysis and display of genome-wide expression patterns Proc Natl Acad Sci USA 1998 95 25 14863 14868 10.1073/pnas.95.25.14863 9843981 PMC24541

[B60] Saldanha AJ Java Treeview–extensible visualization of microarray data Bioinformatics 2004 20 17 3246 3248 10.1093/bioinformatics/bth349 15180930

[B61] Trapnell C Pachter L Salzberg SL TopHat: discovering splice junctions with RNA-Seq Bioinformatics 2009 25 9 1105 1111 10.1093/bioinformatics/btp120 19289445 PMC2672628

[B62] Trapnell C Hendrickson DG Sauvageau M Goff L Rinn JL Pachter L Differential analysis of gene regulation at transcript resolution with RNA-seq Nat Biotechnol 2013 31 1 46 53 23222703 10.1038/nbt.2450 PMC3869392

