# Supplementary figures and images for: Polysome profiling reveals broad translatome remodeling during endoplasmic reticulum (ER) stress in the pathogenic fungus Aspergillus fumigatus
Source: BMC Genomics. 2014 Feb 25;15:159. doi: 10.1186/1471-2164-15-159 (PMC3943501; doi:10.1186/1471-2164-15-159)

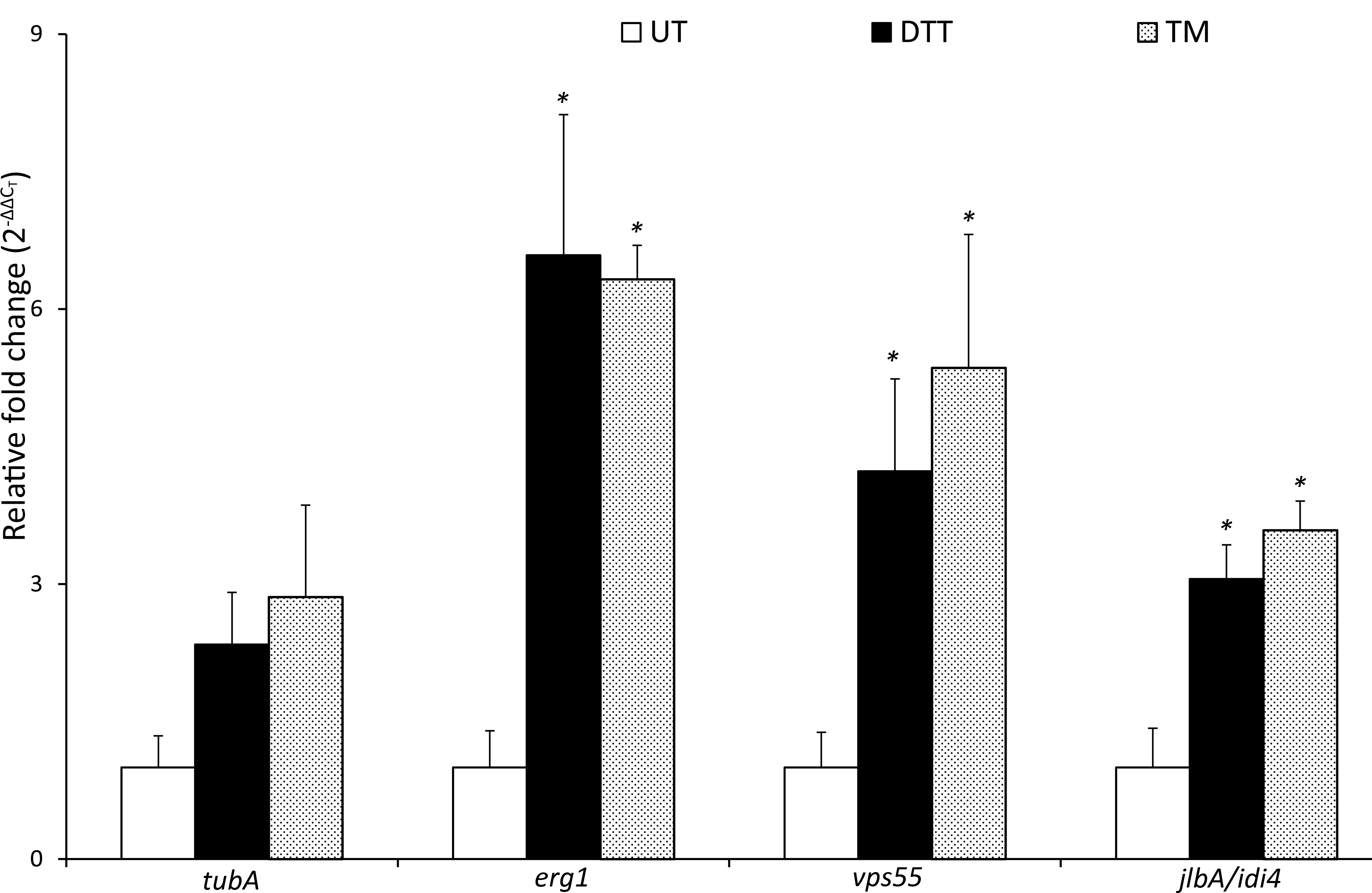

Supplement: Additional file 1 — Validation of the translationally regulated dataset by qPCR. The levels of 18S rRNA in fraction-U or fraction-W was used as an endogenous control to derive a ∆Ct value for each fraction. A translational efficiency ratio (W/U) was then calculated by subtracting ∆Ct of fraction-W from that of fraction-U, representing ∆∆Ct. The change in W/U ratios upon treatment with DTT or TM was then plotted using 2-∆∆Ct of untreated samples (UT) as the reference. [file 1471-2164-15-159-S1.tiff]
