# Supplementary material for: Polysome profiling reveals broad translatome remodeling during endoplasmic reticulum (ER) stress in the pathogenic fungus Aspergillus fumigatus
Source: BMC Genomics. 2014 Feb 25;15:159. doi: 10.1186/1471-2164-15-159 (PMC3943501; doi:10.1186/1471-2164-15-159)
Supplement: Additional file 2 — List of mRNAs with decreased polysome association during ER stress (treatment with DTT or TM). Values represent log2 [translational state efficiency], as described in Methods. [file 1471-2164-15-159-S2.docx]

**Table S2.** List of mRNAs with decreased polysome association during ER stress (treatment with DTT or TM). Values represent log2[translational state efficiency], as described in Methods.

| DTT | TM | mRNA |
| --- | --- | --- |
| Metabolism | | |
| -2.21 | -1.23 | short-chain dehydrogenase (AFUA_4G00160) |
| -2.14 | -1.55 | cytochrome c mitochondrial import factor (Cyc2), putative (AFUA_6G04980) |
| -2.01 | -1.14 | GMC oxidoreductase (AFUA_3G08070) |
| -2.01 | -1.08 | N-acylethanolamine amidohydrolase (AFUA_1G14880) |
| -1.35 | -1.08 | aldehyde dehydrogenase (AFUA_4G08600) |
| -1.33 | -1.82 | lysophospholipase Plb2 (AFUA_5G01340) |
| -1.31 | -1.12 | DRAP deaminase (Rib2) (AFUA_2G16360) |
| -1.30 | -2.00 | ribose phosphate diphosphokinase Prs1 (AFUA_7G05670) |
| -1.30 | -1.14 | branched-chain amino acid aminotransferase (AFUA_1G01680) |
| -1.07 | -2.34 | FAD binding monooxygenase (AFUA_7G00270) |
| -1.01 | -2.12 | acid phosphatase (AFUA_1G16480) |
| RNA processing | | |
| -2.39 | -1.21 | RNA polymerase II Elongator subunit (AFUA_6G05090) |
| -1.89 | -1.15 | pre-rRNA processing protein (AFUA_6G04590) |
| -1.63 | -2.41 | cofactor for methionyl- and glutamyl-tRNA synthetases, putative (AFUA_2G15940) |
| -1.61 | -1.01 | rRNA processing protein (Ebp2) (AFUA_5G12270) |
| -2.06 | -2.41 | DEAD box helicase Mph1 (AFUA_1G03050) |
| -1.01 | -1.22 | mRNA cleavage factor complex II protein Clp1 (AFUA_5G11400) |
| Transcription factors | | |
| -1.62 | -1.89 | C6 transcription factor (AFUA_3G15290) |
| Cell cycle | | |
| -1.25 | -1.19 | cell division control protein Cdc48 (AFUA_2G17110) |
| -1.69 | -1.61 | cell cycle control protein (Cwf19) (AFUA_4G11830) |
| -1.51 | -1.47 | DNA damage and replication checkpoint protein Rfx1, putative (AFUA_5G06120) |
| Others | | |
| -2.34 | -1.87 | TAM domain methyltransferase (AFUA_2G04890) |
| -2.48 | -1.06 | short chain oxidoreductase/dehydrogenase (AFUA_1G11010) |
| -3.00 | -2.17 | Kelch repeats protein (AFUA_2G04970) |
| -2.06 | -1.07 | dienelactone hydrolase family protein (AFUA_4G01130) |
| -2.04 | -1.01 | acyltransferase (AFUA_6G14030) |
| -1.97 | -1.17 | casein kinase (AFUA_8G04810) |
| -1.92 | -1.31 | conserved glutamic acid-rich protein (AFUA_1G15870) |
| -1.83 | -1.75 | Phosphotransferase enzyme family domain protein (AFUA_1G02880) |
| -1.79 | -2.09 | extracellular GDSL-like lipase/acylhydrolase (AFUA_2G00820) |
| -1.63 | -1.70 | nuclear pore complex subunit Nup159 (AFUA_2G04920) |
| -1.62 | -1.05 | glyoxalase family protein (AFUA_3G06020) |
| -1.56 | -2.43 | ubiquitin C-terminal hydrolase (AFUA_6G12270) |
| -1.55 | -1.56 | MFS monocarboxylic acid transporter (AFUA_5G13230) |
| -1.52 | -1.24 | integral membrane protein (AFUA_6G00320) |
| -1.50 | -1.97 | phospholipase PldA (AFUA_7G05580) |
| -1.44 | -2.79 | histone ubiquitinationc protein (Bre1) (AFUA_6G04390) |
| -1.43 | -1.39 | RTA1 domain protein (AFUA_6G14140) |
| -1.37 | -1.77 | extracellular dioxygenase (AFUA_6G03070) |
| -1.36 | -1.68 | oligopeptide transporter, OPT family (AFUA_1G13620) |
| -1.30 | -1.43 | kinesin family protein (AFUA_5G13050) |
| -1.26 | -1.15 | peroxidase (AFUA_5G02300) |
| -1.26 | -2.75 | GTP-binding protein Sey1 (AFUA_1G12180) |
| -1.24 | -1.09 | C6 finger domain protein (AFUA_2G17511) |
| -1.22 | -1.68 | MYB DNA-binding domain protein (AFUA_1G07560) |
| -1.19 | -2.46 | karyopherin alpha subunit (AFUA_2G16090) |
| -1.18 | -1.56 | nucleolus protein required for cell viability (AFUA_1G09320) |
| -1.14 | -1.10 | endoglucanase (AFUA_3G03950) |
| -1.13 | -1.06 | kinesin family protein (KipA) (AFUA_7G03710) |
| -1.11 | -2.24 | C2H2 finger domain protein (AFUA_6G12020) |
| -1.10 | -2.25 | cAMP-dependent protein kinase pathway protein (Som1), putative (AFUA_7G02260) |
| -1.10 | -1.09 | WD repeat-containing protein (AFUA_1G02990) |
| -1.10 | -2.25 | phenol 2-monooxygenase (AFUA_6G03490) |
| -1.07 | -1.15 | actin-related protein RO7 (AFUA_3G13250) |
| -1.01 | -1.12 | GPI anchored cell wall protein (AFUA_3G01150) |
| Hypothetical proteins | | |
| -3.27 | -1.27 | hypothetical protein (AFUA_3G14870) |
| -3.04 | -1.73 | hypothetical protein (AFUA_1G00660) |
| -2.43 | -1.03 | hypothetical protein (AFUA_5G01720) |
| -2.28 | -2.04 | hypothetical protein (AFUA_4G04650) |
| -2.13 | -1.38 | hypothetical protein (AFUA_6G09570) |
| -2.07 | -1.27 | hypothetical protein (AFUA_3G00960) |
| -2.05 | -1.02 | hypothetical protein (AFUA_3G02450) |
| -2.04 | -1.19 | hypothetical protein (AFUA_7G08260) |
| -1.95 | -1.23 | hypothetical protein (AFUA_1G15660) |
| -1.88 | -2.24 | hypothetical protein (AFUA_3G05640) |
| -1.84 | -1.02 | hypothetical protein (AFUA_7G01990) |
| -1.79 | -1.68 | hypothetical protein (AFUA_4G06590) |
| -1.72 | -3.06 | hypothetical protein (AFUA_8G06270) |
| -1.61 | -1.45 | hypothetical protein (AFUA_4G00490) |
| -1.56 | -1.74 | hypothetical protein (AFUA_3G07350) |
| -1.53 | -1.46 | hypothetical protein (AFUA_1G00700) |
| -1.51 | -1.75 | hypothetical protein (AFUA_6G09315) |
| -1.43 | -2.42 | hypothetical protein (AFUA_4G13980) |
| -1.41 | -2.00 | hypothetical protein (AFUA_7G00880) |
| -1.34 | -1.84 | hypothetical protein (AFUA_4G08090) |
| -1.29 | -1.53 | hypothetical protein (AFUA_1G09720) |
| -1.26 | -1.01 | hypothetical protein (AFUA_1G15540) |
| -1.13 | -1.85 | hypothetical protein (AFUA_8G02670) |
| -1.12 | -1.10 | hypothetical protein (AFUA_1G01410) |
| -1.11 | -1.02 | hypothetical protein (AFUA_8G01330) |
| -1.11 | -1.66 | hypothetical protein (AFUA_3G05960) |
| -1.07 | -2.13 | hypothetical protein (AFUA_2G10830) |
| -1.06 | -1.28 | hypothetical protein (AFUA_6G11710) |
| -1.04 | -1.87 | hypothetical protein (AFUA_4G14170) |
| -1.04 | -1.52 | hypothetical protein (AFUA_6G02360) |
| -1.03 | -2.69 | hypothetical protein (AFUA_2G14547) |
| -1.02 | -1.99 | hypothetical protein (AFUA_5G14110) |
| -1.02 | -1.12 | hypothetical protein (AFUA_5G03150) |
| -1.01 | -1.84 | hypothetical protein (AFUA_4G09770) |
