# Supplementary material for: Polysome profiling reveals broad translatome remodeling during endoplasmic reticulum (ER) stress in the pathogenic fungus Aspergillus fumigatus
Source: BMC Genomics. 2014 Feb 25;15:159. doi: 10.1186/1471-2164-15-159 (PMC3943501; doi:10.1186/1471-2164-15-159)
Supplement: Additional file 3 — List of over-represented KEGG pathways in the dataset of translationally regulated mRNA following a shift to 37°C. [file 1471-2164-15-159-S3.docx]

| **KEGG pathway** | ***p-value*** |
| --- | --- |
| **Early thermal stress** | |
| N-Glycan biosynthesis | 1.40E-02 |
| DNA replication | 1.79E-02 |
| **Continuous thermal stress** | |
| Ribosome | 1.79E-03 |
| Oxidative phosphorylation | 4.91E-02 |
| **Late thermal stress** | |
| Alanine, aspartate and glutamate metabolism | 1.31E-03 |
| Oxidative phosphorylation | 1.34E-02 |
| Spliceosome | 2.13E-02 |
| Phosphatidylinositol signaling system | 3.32E-02 |
| Citrate cycle (TCA cycle) | 4.42E-02 |
| Taurine and hypotaurine metabolism | 4.55E-02 |
| **ER stress (DTT & TM)** | |
| Pantothenate and CoA biosynthesis | 7.80E-03 |
| Glutathione metabolism | 2.66E-02 |
| Oxidative phosphorylation | 2.93E-02 |
|  |  |

**Additional file 3: List of over-represented KEGG pathways in the dataset of translationally regulated mRNA following a shift to 37^o^C.**
