# Supplementary material for: Polysome profiling reveals broad translatome remodeling during endoplasmic reticulum (ER) stress in the pathogenic fungus Aspergillus fumigatus
Source: BMC Genomics. 2014 Feb 25;15:159. doi: 10.1186/1471-2164-15-159 (PMC3943501; doi:10.1186/1471-2164-15-159)
Supplement: Additional file 4 — List of mRNAs with increased polysome association during each of the three forms of ER stress: treatment with DTT, TM and thermal stress. Values represent log2[translational efficiency ratio], as described in Methods. #mRNAs subject to translational upregulation in the thermal stress dataset at 60 min. [file 1471-2164-15-159-S4.docx]

**Additional file 4: List of mRNAs with increased polysome association during each of the three forms of ER stress: treatment with DTT, TM and thermal stress. Values represent log2[translational efficiency ratio], as described in Methods. ^#^ mRNAs subject to translational upregulation in the thermal stress dataset at 60 min.**

| DTT | TM | 37°C^#^ | Gene |
| --- | --- | --- | --- |
| 2.90 | 1.62 | 1.02 | glycosyl transferase (AFUA_8G02070) |
| 1.95 | 3.92 | 1.09 | hypothetical protein (AFUA_2G11860) |
| 1.83 | 2.58 | 1.10 | disulfide isomerase (TigA) (AFUA_5G12260) |
| 1.05 | 1.23 | 1.14 | hypothetical protein (AFUA_8G05120) |
| 1.01 | 2.42 | 1.18 | 40S ribosomal protein Rps16 (AFUA_2G10500) |
| 1.26 | 1.73 | 1.19 | short chain dehydrogenase/reductase family protein (AFUA_6G04120) |
| 2.11 | 2.89 | 1.23 | prefoldin subunit 5 (AFUA_1G10740) |
| 1.07 | 3.32 | 1.28 | hypothetical protein (AFUA_8G02540) |
| 1.05 | 2.33 | 1.31 | transformer-SR ribonucleoprotein (AFUA_7G05260) |
| 2.28 | 1.59 | 1.34 | F-box domain protein (AFUA_2G02570) |
| 1.57 | 3.04 | 1.36 | RAB GTPase Vps21/Ypt51 (AFUA_3G10740) |
| 4.51 | 2.08 | 1.39 | aconitate hydratase (AFUA_3G08080) |
| 1.24 | 1.99 | 1.42 | general stress response phosphoprotein phosphatase Psr1/2 (AFUA_1G04790) |
| 1.63 | 3.90 | 1.43 | CHCH domain protein (AFUA_3G06370) |
| 3.09 | 1.71 | 1.43 | bZIP transcription factor (LziP) (AFUA_1G16460) |
| 1.32 | 2.63 | 1.44 | hypothetical protein (AFUA_5G07480) |
| 2.00 | 1.25 | 1.51 | ubiquitin conjugating enzyme (UbcC) (AFUA_5G09200) |
| 1.93 | 1.75 | 1.52 | Mitochondrial import inner membrane translocase subunit (TIM22) (AFUA_5G02200) |
| 1.10 | 1.27 | 1.57 | mitochondrial deoxynucleotide carrier protein (AFUA_2G14980) |
| 1.47 | 1.51 | 1.61 | Leucine Rich Repeat domain protein (AFUA_4G11700) |
| 2.36 | 2.77 | 1.65 | opsin (AFUA_7G01430) |
| 1.40 | 1.17 | 1.67 | hypothetical protein (AFUA_3G01590) |
| 1.67 | 1.40 | 1.68 | cell wall protein (AFUA_3G10960) |
| 1.85 | 3.11 | 1.75 | flavin dependent monooxygenase (AFUA_5G03380) |
| 2.57 | 1.77 | 1.83 | mitotic spindle biogenesis protein Spc19 (AFUA_2G02280) |
| 1.06 | 2.49 | 1.83 | hypothetical protein (AFUA_4G10440) |
| 1.97 | 1.35 | 1.90 | Mago nashi domain protein (AFUA_1G10960) |
| 1.15 | 2.72 | 2.06 | RING finger domain protein (AFUA_3G12190) |
| 1.10 | 2.58 | 2.14 | integral membrane protein (Pth11) (AFUA_6G03600) |
| 3.40 | 2.04 | 2.16 | selenoprotein domain protein (AFUA_1G05220) |
| 1.05 | 2.99 | 2.18 | class II aldolase/adducin domain protein (AFUA_4G12840) |
| 1.55 | 1.87 | 2.29 | NADH-ubiquinone oxidoreductase subunit GRIM-19, putative (AFUA_3G08770) |
| 5.38 | 2.13 | 2.30 | Vacuolar protein sorting 55 superfamily (AFUA_6G04780) |
| 1.13 | 1.57 | 2.35 | hypothetical protein (AFUA_1G11535) |
| 3.83 | 2.98 | 2.40 | BolA domain protein (AFUA_7G01520) |
| 2.50 | 1.69 | 2.43 | oleate delta-12 desaturase (AFUA_1G12530) |
| 1.11 | 2.60 | 2.52 | calcineurin binding protein (AFUA_2G13060) |
| 2.25 | 1.35 | 2.62 | allergen Asp F7 (AFUA_4G06670) |
| 1.51 | 1.93 | 2.87 | 40S ribosomal protein S9 (AFUA_3G06970) |
| 2.61 | 1.10 | 3.09 | hypothetical protein (AFUA_4G03010) |
| 3.07 | 3.04 | 3.15 | bZIP transcription factor JlbA/IDI-4 (AFUA_5G01650) |
| 2.23 | 2.62 | 3.29 | NADH-ubiquinone oxidoreductase 304 kDa subunit precursor (AFUA_6G08810) |
| 2.87 | 2.26 | 3.59 | 40S ribosomal protein S8 (AFUA_6G07360) |
| 4.83 | 1.49 | 4.13 | small nuclear ribonucleoprotein SmD1 (AFUA_2G05110) |
